# Associations between dietary patterns and sarcopenia: insights from cross-sectional and Mendelian randomization analyses

**DOI:** 10.1080/15502783.2025.2564239

**Published:** 2025-09-22

**Authors:** Jiale Tan, Xinyi Chen, Zhen Peng, Hongyu Bai, Luze Shi, Yaying Sun, Jiwu Chen

**Affiliations:** Department of Sports Medicine, Shanghai General Hospital, Shanghai Jiao Tong University School of Medicine, Shanghai Jiao Tong University, Shanghai, China

**Keywords:** Sarcopenia, dietary patterns, cross sectional study, UK Biobank, Mendelian randomization analysis

## Abstract

**Background:**

Sarcopenia is defined as a progressive and generalized skeletal muscle disorder characterized by the accelerated loss of muscle mass and function. Current studies have revealed the significant impact of some dietary factors on the development and progression of sarcopenia. However, these studies often focused on single nutrients but little had examined complete dietary strategies. Thus, comprehensive evaluations of diverse dietary factors in larger populations are crucial for effectively adjusting diets to delay sarcopenia progression, enhancing quality of life and independence in the elderly.

**Methods:**

We conducted a cross-sectional analysis to assess the potential correlation between various dietary factors and sarcopenia based on the population data from the UK Biobank. This analysis employed multivariate logistic regression models adjusted for different covariates (demographic data including age, sex, ethnics, etc., and potential confounders including smoking status, activity group, body mass index, etc.). Furthermore, to evaluate the robustness of our findings, we performed sub-analyses and calculated the Variance Inflation Factor. To investigate the causal influence of dietary factors on sarcopenia-related phenotypes, including low hand grip strength and appendicular lean mass (ALM), we conducted a Mendelian Randomization (MR) analysis. The analytic methods of MR were selected based on the outcomes of sensitivity analyses.

**Results:**

In the cross-sectional analysis, a total of 211,027 participants were included in the final analysis, with a mean age of 56 years, comprising 104,271 females and 106,756 males. We identified significant correlations between the frequency and type of food intake and sarcopenia. For probable sarcopenia diagnosed by grip strength, a higher intake frequency of oily fish is negatively correlated with sarcopenia risk, whereas processed meat shows a positive correlation. Preference for FloraPro-Active/Benecol in spreads also relates to a reduced risk. Preliminary findings indicate correlations between tea (OR = 1.02, *p* < 0.01), coffee (OR = 1.02, *p* < 0.01), fresh fruit intake (OR = 1.01, *p* < 0.01), and probable sarcopenia. For confirmed sarcopenia diagnosed by grip strength and ALM, a higher intake frequency of processed meat could increase sarcopenia risk and preference for Bran cereal in cereal type and wholemeal or wholegrain in bread type also related to a reduced risk. The MR analysis revealed that coffee (ground coffee (OR = 3.44, *p* < 0.0035), instant coffee (OR = 2.69, *p* < 0.015), decaffeinated coffee (OR = 3.94, *p* < 0.0004)) intake significantly increased the risk of low grip strength, while water (OR = 1.06, *p* < 0.000398) and fruit (OR = 1.10, *p* < 0.0065) intake enhance ALM. Conversely, psychoactive drinks (OR = 0.91, *p* < 0.00001), alcohol (OR = 0.906, *p* < 0.0012), and decaffeinated coffee (OR = 0.65, *p* < 0.0047) decreased ALM. These results have undergone sensitivity analysis validation.

**Conclusions:**

Our analysis using UK Biobank data explored associations between dietary factors and sarcopenia. We found strong links between probable sarcopenia and the intake of oily fish, processed meat, lamb, and coffee, whereas processed meat remained significantly related with confirmed sarcopenia. Preference for specific foods, such as FloraPro-Active/Benecol spreads, Bran cereal and wholemeal or wholegrain bread were associated with reduced risk. MR confirmed the causal effects of coffee intake on low hand grip strength and ALM. Our study provides insights for dietary strategies in sarcopenia patients, though further research is needed to validate and explore these mechanisms.

## 1. Introduction

Sarcopenia is defined as a progressive and generalized skeletal muscle disorder characterized by the accelerated loss of muscle mass and function [1,2]. It is primarily associated with aging but can also be influenced by other factors such as inactivity, malnutrition, and chronic diseases [3–6]. Epidemiologically, sarcopenia is a prevalent condition affecting approximately 5–13% of adults aged 60–70 years and up to 50% of those aged over 80 years [7]. The condition poses a significant public health challenge due to its association with adverse outcomes, including physical disability, decreased quality of life, and increased mortality [8,9].

Research into sarcopenia has expanded considerably over recent decades, focusing on identifying the underlying mechanisms, risk factors, and effective interventions for prevention and treatment. Current studies suggest that the pathophysiology of sarcopenia involves various factors, including hormonal changes, inflammation, and insulin resistance [10,11]. Additionally, the significant impact of dietary factors on the development and progression of sarcopenia has gradually attracted the interest of researchers. Nutritional status plays a crucial role in muscle health, and inadequate intake of macro- and micro-nutrients has been associated with increased risk of muscle mass decline and functional impairment [12]. For example, the association between total calories intake and sarcopenia has been extensively studied. Mean caloric intake has been found to be significantly lower in individuals with sarcopenia compared to those without the condition. Furthermore, protein intake was suggested that adequate and high-quality protein consumption can help preserve muscle mass and strength in older adults [13,14]. Moreover, vitamin D, omega-3 fatty acids, and antioxidants have also been identified as potentially beneficial in combating sarcopenia [15–18]. Despite these insights, these studies are usually limited to single nutrients, thus providing limited value in guiding dietary choices for individuals with sarcopenia. Therefore, evaluating dietary factors in larger populations can help optimize nutrition for sarcopenia patients, potentially delaying disease progression and improving their quality of life and independence.

The UK Biobank is a prospective cohort comprising more than 500,000 individuals aged 40–69 years recruited between 2006 and 2010 from across the UK [19]. Participants completed detailed questionnaires on demographics, health history, and lifestyle, and provided physical measurements (e.g. height, weight, blood pressure, grip strength) and biological samples (blood, urine, saliva) [20,21]. Given that the database contains comprehensive dietary factors, demographic questionnaire data, and sarcopenia-related phenotypes (grip strength and appendicular lean mass(ALM)), we decided to utilize population data from the UK Biobank to examine the association between various dietary factors and sarcopenia.

However, since cross-sectional studies can only identify associations between dietary factors and sarcopenia without establishing causality [22,23], we conducted a Mendelian randomization (MR) analysis to investigate the causal relationship between dietary factors and sarcopenia-related phenotypes. By using genetic variants as instrumental variables to infer causal relationships, MR analysis could address reverse causation and residual confounding – key limitations of traditional observational studies [16]. Thus, it has been successfully employed to elucidate risk factors for a wide range of diseases, like coronary heart disease and Alzheimer’s disease [24,25].

Therefore, this study performed a cross-sectional analysis using UK Biobank population data to examine the association between dietary patterns and probable sarcopenia. To further validate the potential causal relationship between dietary factors and sarcopenia, we conducted a MR analysis utilizing Genome-wide association study (GWAS) data of dietary factors and sarcopenia-related phenotypes, including hand grip strength and appendicular lean mass.

## 2. Methods

### 2.1. Data source and study population

UK Biobank is a large prospective cohort study that has enrolled over 500,000 participants, aged 37 to 73, from 22 assessment centers across the United Kingdom [26]. We extracted baseline data (2006–2010) from the UK Biobank, which includes comprehensive information on participants’ dietary factors, grip strength, as well as demographic, socioeconomic, and lifestyle factors. Initially, we retrieved complete data for 502,377 participants from the UK Biobank. In subsequent analyses, 291,350 participants were excluded due to missing phenotypic data, resulting in a final sample of 211,027 participants included in the analysis.

GWAS outcome data (curated statistical results that quantify the strength of association between genetic variants (typically single-nucleotide polymorphisms, SNPs) and a specific trait or disease phenotype in a Genome-wide association study) on dietary factors were obtained by Pirastu et al [27]. This study included a total of 445,779 participants and identified 283 genetic markers associated with dietary factors. Subsequently, they converted these associations into direct genetic effects on food exposures by adjusting them for effects mediated via other traits. This study created comprehensive map of the genetic determinants of dietary factors in the UK Biobank. GWAS summary data of appendicular lean mass (ALM) was sourced from Pei et al [28]. They performed GWAS analysis in 450,243 participants (including 244,730 females and 205,513 males) targeting ALM and identified 1059 conditionally independent variants from 799 loci. GWAS outcome data of low hand grip strength were sourced from Jones et al’s study, which involved 256,523 participants from a European population and identified people with low grip strength according to two definitions: European Working Group on Sarcopenia in Older People (EWGSOP: grip strength < 30 kg Male; < 20 kg Female) and FNIH sarcopenia project 2014 (grip strength < 26 kg Male; < 16 kg Female) [29]. Two GWAS outcome datasets, based on these two criteria, were used for MR analysis with dietary factors, respectively.

### 2.2. Ethical approval

The UK Biobank was approved by the North West Multi-Center Research Ethics Committee with all participants provided written informed consent. Specific protocol is publicly available at (http://www.ukbiobank.ac.uk/). We applied to obtain the population data related to UK Biobank for this study under the application number 95,082.

### 2.3. Definition of sarcopenia

According to the EWGSOP2 criteria [1], probable sarcopenia is defined as a maximum handgrip strength (HGS) of less than 27 kg in males and less than 16 kg in females. The HGS was recorded for each hand, and the average value was used in our research. Confirmed sarcopenia is diagnosed when, in addition to meeting the criteria for probable sarcopenia, the following condition is present: ALM/height^2^ (ALM index, ALMI) < 7.0 kg/m^2^ in males and < 5.5 kg/m^2^ in females. The Jamar J00105 hydraulic handheld dynamometer was used to measure HGS among UK Biobank participants.

### 2.4. Assessments of dietary factors

The participants’ dietary factors were assessed using a touchscreen questionnaire in the assessment center. The diets that were finally analyzed as continuous variables included cooked vegetable intake, salad/raw vegetable intake, Fresh fruit intake, dried fruit intake, bread intake, tea intake, coffee intake and water intake. Among them, cooked vegetable intake and salad/raw vegetable intake were defined as the average number of heaped tablespoons consumed per day. Fresh fruit and dried fruit were defined as average pieces consumed per day. Tea, coffee and water intake were defined as average cups consumed every day. Bread intake was defined as average slices consumed per week. Cereal intake was defined as the average bowls of cereal consumed every week. The remaining dietary factors were included as categorical variables in subsequent analysis. Among them, oily fish, non oily fish, processed meat, poultry, beef, lamb mutton, pork and cheese intake were all divided into six groups: 2–4 times a week, 5–6 times a week, less than once a week, never, once a week, once or more daily. Milk type used was divided into six groups: full cream, never/rarely have milk, other type of milk, semi-skimmed, skimmed, soya milk. Spread type was divided into four groups: butter/spreadable butter, flora pro-active/benecol, never/rarely use spread, other type of spread/margarine. Bread type was divided into four groups: brown, other type of bread, white, wholemeal or wholegrain. Salt added to food was divided into four groups: always, never/rarely, sometimes, usually. Cereal type was divided into five groups: Biscuit cereal, oat cereal, bran cereal, muesli and other. Coffee type was divided into four groups: Instant coffee, ground coffee, decaffeinated coffee, and other types of coffee.

### 2.5. Assessments of covariates

Demographic data including age, sex and ethnic background were collected during the initial visit to the assessment center.

Townsend deprivation index (an area-level measure of material deprivation) is derived from census data. It combines four standardized indicators: unemployment, household overcrowding, lack of car ownership, and non-homeownership. The composite score reflects relative deprivation, with higher positive values indicating greater deprivation and negative values suggesting affluence) was calculated immediately prior to participant joining UK Biobank based on the preceding national census output areas. Each participant is assigned a score corresponding to the output area in which their postcode is located. Average total household income before tax was obtained from the touchscreen questionnaire and divided into five groups: <£18000, £18000–30999, £31000–51999, £52000–100000, and >£100000. Qualifications of education were divided into four groups: college, high school, others, none of the above. Participants’ self-reported physical activity intensity was assessed using International Physical Activity Questionnaire (IPAQ) and was divided into three groups: low, moderate, and high. Participants who met either of the following criteria would be classified as moderate: a) 3 or more days of vigorous-intensity activity of at least 20 min per day; b) 5 or more days of moderate-intensity activity and/or walking of at least 30 min per day; c) 5 or more days of any combination of walking, moderate-intensity or vigorous intensity activities achieving a minimum total physical activity of at least 600 MET-minutes/week. Participants who met either of the following criteria would be classified as high a) vigorous-intensity activity on at least 3 days achieving a minimum total physical activity of at least 1500 MET-minutes/week; b) at least 7 days of walking, moderate-intensity, or vigorous-intensity activity, totaling ≥3000 MET-minutes/week. Smoking status was divided into three groups: Never, previous and current. Alcohol intake frequency was divided into six groups: daily or almost daily, never, once or twice a week, one to three times a month, special occasions only, three or four times a week.

### 2.6. Statistical analysis

Multivariable logistic regression models [30] were employed to explore the relationship between various dietary factors and probable sarcopenia diagnosed via handgrip strength (HGS). The association was quantified using odds ratios (OR) with 95% confidence intervals, derived from the regression analysis. To thoroughly and rigorously evaluate the diet-sarcopenia association, three models were constructed: Model 1 included dietary factors and demographic variables, such as age, sex, ethnic background, Townsend deprivation index, average household income, educational qualifications, and alcohol intake frequency; Model 2 expanded on Model 1 by adding lifestyle factors including IPAQ groups, smoking status; Model 3 further included body mass index (BMI), weight, and standing height. *p* < 0.05 is considered as a statistically significant threshold. We rounded the P-values to two decimal places in the regression analysis results while all P-values less than 0.01 were uniformly recorded as < 0.01.

To assess the multicollinearity among the predictor variables in the logistic regression model, we calculated the Variance Inflation Factor (VIF) [31] for each independent variable. The VIF quantifies the extent of correlation among predictors, which can inflate the variance of the coefficient estimates and affect model stability. Furthermore, the VIF was adjusted for multivariate variables’ degrees of freedom (GVIF^(1/(2*Df))). The adjusted VIF values exceeding 10 were generally considered indicative of significant multicollinearity, warranting further investigation. By systematically evaluating these metrics, we ensured the robustness of our model interpretations, minimizing the risk of biased coefficient estimates due to collinear predictors.

For sensitivity analysis, we conducted a subsample analysis to assess the robustness of our logistic regression model results. Specifically, we randomly selected 80% of our original dataset to create a subsample. Utilizing the same logistic regression formula as applied to the model 3 in full dataset, we re-estimated the model on this subsample. This approach allows for the evaluation of the model’s stability and consistency across different samples of the data. By comparing the coefficients and overall fit between the full data model and the subsample model, we can ascertain the sensitivity of our findings to variations in the data composition.

In MR analysis, four methods were employed: inverse-variance weighted (IVW), weighted median, weighted mode, and MR-Egger [32]. Typically, IVW was used to estimate the causal relationship between diet and phenotypes associated with sarcopenia, specifically low handgrip and appendicular lean mass (ALM). A p-value of < 0.05 was considered indicative of a significant causal relationship. In screening for single nucleotide polymorphisms (SNPs), we selected SNPs at the genome-wide significance level (p < 5 × 10 − 8) as instrumental variables employed in MR analysis. Identification criteria of genetic variants were as follows: Linkage disequilibrium threshold was set as 0.001 for linkage disequilibrium parameter (r2) and a genetic distance of 10,000 kb. The linkage disequilibrium parameter (r2) was estimated based on 1000 Genomes European panel. Otherwise, the R^2^ and F-statistics for each SNP were calculated using the following equations: R2 = 2 × (1-MAF) × MAF × Beta2); F = R^2 (*n*-2)/1-R^2. SNPs with F less than 10, which indicates a high risk of bias in weak instrumental variables, were excluded. We ensured no heterogeneity or horizontal pleiotropy in MR estimates, as indicated by a Cochran’s Q p-value ( > 0.05) and MR-Egger intercept p-value (> 0.05) [33]. The selection of methods for MR analysis is based on the following principles:1. In the absence of heterogeneity and pleiotropy, the estimated results from the IVW method were preferred. 2. In the presence of heterogeneity but without pleiotropy, the results from the Weighted Median method were preferred (the random effects model of IVW can also be employed). 3. In situations where pleiotropy was present, the results calculated using the MR-Egger method were preferred. Finally, false discovery rate (FDR) approach was used to correct for multiple testing, as a FDR value < 0.05 was considered significant. MR analyses were conducted using the TwoSampleMR package in R software (version 4.4.1).

## 3. Results

### 3.1. Demographic characteristics

A total of 211,027 participants were finally included in the study. Among them, 14,607 were classified as probable sarcopenia based on the diagnostic criteria for grip strength, while the remaining participants were classified as no sarcopenia. The cohort comprised 106,756 males and 104,271 females, with an average age of 56 years. Additionally, the probable sarcopenia group have had a lower proportion of males, as well as lower average annual household income, educational level, physical activity, height, weight, and grip strength in both hands compared to the non-sarcopenia group. However, the sarcopenia group exhibited a higher BMI. Detailed baseline demographic data are displayed in Table 1.

**Table 1.** Demographic characteristics of participants (*n* = 211,027).

Characteristics	levels	No sarcopenia (N = 196420)	Probable sarcopenia (N = 14607)	p
Age	Mean ± SD	56.12 (8.04)	59.99 (7.04)	< .001
Sex (%)				
	Female	95448 (48.6)	8823 (60.4)	< .001
	Male	100972 (51.4)	5784 (39.6)	
Townsend deprivation index	Mean ± SD	−1.71 (2.84)	−1.15 (3.08)	< .001
Average total household income (%)				
	18,000 to 30,999	48678 (24.8)	4245 (29.1)	< .001
	31,000 to 51,999	54096 (27.5)	3156 (21.6)	
	52,000 to 100,000	45810 (23.3)	1769 (12.1)	
	Greater than 100,000	12825 (6.5)	420 (2.9)	
	Less than 18,000	35011 (17.8)	5017 (34.3)	
Education (%)				
	College	77795 (39.6)	4373 (29.9)	< .001
	High school	24190 (12.3)	1566 (10.7)	
	None of the above	21469 (10.9)	2932 (20.1)	
	Others	72966 (37.1)	5736 (39.3)	
Ethnic background (%)				
	Asian	1947 (1.0)	418 (2.9)	< .001
	Black	606 (0.3)	52 (0.4)	
	Others	2220 (1.1)	203 (1.4)	
	White	191647 (97.6)	13934 (95.4)	
IPAQ activity group (%)				
	high	80337 (40.9)	5008 (34.3)	< .001
	low	34175 (17.4)	3327 (22.8)	
	moderate	81908 (41.7)	6272 (42.9)	
Smoking status (%)				
	Current	17310 (8.8)	1274 (8.7)	.147
	Never	108963 (55.5)	7999 (54.8)	
	Previous	70147 (35.7)	5334 (36.5)	
BMI	Mean ± SD	27.21 (4.50)	27.81 (5.06)	< .001
Weight	Mean ± SD	78.94 (15.54)	76.07 (15.75)	< .001
Height	Mean ± SD	170.09 (9.20)	165.22 (9.01)	< .001
Hand grip strength (left)	Mean ± SD	32.18 (10.70)	15.21 (6.69)	< .001
Hand grip strength (right)	Mean ± SD	34.34 (10.69)	17.42 (6.81)	< .001

BMI: body mass index, IPAQ activity group: International Physical Activity Questionnaire group.

### 3.2. Associations between dietary factors and sarcopenia in the UK Biobank cohorts

Significant relationships between dietary factors with probable sarcopenia were observed in the logistic regression model, which were summarized in Tables 2–4. In model 1, the OR value and 95%CI of food intake frequency significantly associated with sarcopenia were as follows (reference: never): oily fish intake (Less than once a week: 0.88, 0.82–0.94, *p* < 0.01; Once a week: 0.87, 0.81–0.94, *p* < 0.01; 2–4 times a week: 1.18, 1.09–1.28, *p* < 0.01; 5–6 times a week: 0.77, 0.61–0.98, *p* = 0.04), processed meat intake (Once a week: 1.14, 1.05–1.25, *p* < 0.01; 2–4 times a week: 1.17, *p* < 0.01, 1.07–1.28; 5–6 times a week: 1.22, *p* < 0.01, 1.06–1.39, *p* < 0.01; Once or more daily: 1.38, 1.10–1.74, *p* = 0.01), beef intake (2–4 times a week: 0.87, 0.79–0.95, *p* < 0.01), lamb mutton intake (2–4 times a week: 1.24, 1.10–1.40, *p* < 0.01), pork intake (Less than once a week: 0.87, 0.82–0.92, *p* < 0.01; Once a week: 0.93, 0.87–1.00, *p* = 0.04; 5–6 times a week: 0.29, 0.09–0.93, *p* = 0.04; Once or more daily: 3.65, 1.66–8.04, *p* < 0.01), cheese intake (2–4 times a week: 0.88, 0.78–0.98, *p* = 0.02; 5–6 times a week: 0.85, 0.75–0.97, *p* = 0.01). The correlation between the preference of different subgroups of the same food type and the risk of sarcopenia in model1 were as follows: Spread type (reference Butter/spreadable butter: Flora Pro-Active/Benecol: 0.39, 0.28–0.56, *p* < 0.01; Other type of spread/margarine: 1.08, 1.04–1.12, *p* < 0.01, Bread type (reference Brown: Other type of bread: 0.87, 0.79–0.96, *p* = 0.01; Wholemeal or wholegrain: 0.85, 0.81–0.9, *p* < 0.01, Salt added to food (reference Always: Never/rarely: 0.90, 0.82–0.98, *p* = 0.02), Coffee type (reference Decaffeinated coffee: Other type of coffee: 1.22, 1.08–1.39, *p* < 0.01), Cereal type (reference Biscuit cereal: Bran cereal: 0.93, 0.88–0.98, *p* = 0.01; Muesli: 0.88, 0.83–0.93, *p* < 0.01; Oat cereal: 0.92, *p* < 0.01, 0.87–0.97; Other: 1.09, 1.03–1.15, *p* < 0.01). Finally, the relationship between the specific weekly intake of some food types and sarcopenia was as follows: Tea intake (1.01, 1.01–1.02, *p* < 0.01), Coffee intake (1.02, 1.01–1.03, *p* < 0.01), Water intake (1.03, 1.02–1.04, *p* < 0.01), Dried fruit intake (0.99, 0.97–1, *p* = 0.01). Compared with model1, the analysis results of some dietary factors in models 2 and 3 have changed to some extent. For example, in model 3, the frequency of pork intake once a week was no longer significant compared with never taking it (model3: 0.95, 0.88–1.02, *p* = 0.12), but sometimes adding salt to food became significant compared with always adding it (model2: 0.91, 0.83–0.99, *p* = 0.04; model 3: 0.90, 0.82–0.99, *p* = 0.03). In addition, compared with Model 1 and 2, the intake of fresh fruit (1.01, 1.00–1.03, *p* = 0.01) and bread (1.00, 1.00–1.01, *p* = 0.02) in Model 3 was found to be significantly related to sarcopenia, while the intake of dried fruits became insignificant. Finally, in models 2 and 3, the frequency of oil fish intake at 5–6 times per week is significantly weaker than that at no time, reaching the threshold marginal level (*p* = 0.05).

**Table 2.** Logistic regression results in model 1.

Variable	OR	95%CI	P
**Oily fish intake (reference: Never)**			
Less than once a week	0.88	0.82, 0.94	< 0.01
Once a week	0.87	0.81, 0.94	< 0.01
2–4 times a week	0.85	0.78, 0.92	< 0.01
5–6 times a week	0.77	0.61, 0.98	0.04
Once or more daily	1.00	0.66, 1.52	1.00
**Non oily fish intake (reference: Never)**			
Less than once a week	0.91	0.82, 1.02	0.10
Once a week	0.92	0.82, 1.02	0.12
2–4 times a week	0.97	0.86, 1.09	0.57
5–6 times a week	0.80	0.58, 1.10	0.17
Once or more daily	1.00	0.56, 1.77	1.00
**Processed meat intake (reference: Never)**			
Less than once a week	1.07	0.98, 1.17	0.12
Once a week	1.14	1.05, 1.25	< 0.01
2–4 times a week	1.17	1.07, 1.28	< 0.01
5–6 times a week	1.22	1.06, 1.39	< 0.01
Once or more daily	1.38	1.10, 1.74	0.01
**Poultry intake (reference: Never)**			
Less than once a week	1.04	0.92, 1.17	0.57
Once a week	0.99	0.88, 1.12	0.92
2–4 times a week	0.98	0.87, 1.11	0.78
5–6 times a week	1.06	0.89, 1.26	0.54
Once or more daily	0.98	0.66, 1.47	0.94
**Beef intake (reference: Never)**			
Less than once a week	1.02	0.94, 1.10	0.70
Once a week	0.97	0.90, 1.06	0.53
2–4 times a week	0.87	0.79, 0.95	< 0.01
5–6 times a week	0.79	0.48, 1.33	0.38
Once or more daily	1.51	0.75, 3.04	0.25
**Lamb mutton intake (reference**: Never)			
Less than once a week	1.12	1.06, 1.20	< 0.01
Once a week	1.20	1.12, 1.29	< 0.01
2–4 times a week	1.24	1.10, 1.40	< 0.01
5–6 times a week	0.92	0.35, 2.42	0.87
Once or more daily	0.46	0.13, 1.70	0.25
**Pork intake (reference: Never)**			
Less than once a week	0.87	0.82, 0.92	< 0.01
Once a week	0.93	0.87, 1.00	0.04
2–4 times a week	1.05	0.94, 1.17	0.37
5–6 times a week	0.29	0.09, 0.93	0.04
Once or more daily	3.65	1.66, 8.04	< 0.01
**Cheese intake (reference: Never)**			
Less than once a week	0.95	0.85, 1.07	0.43
Once a week	0.90	0.80, 1.01	0.06
2–4 times a week	0.88	0.78, 0.98	0.02
5–6 times a week	0.85	0.75, 0.97	0.01
Once or more daily	0.97	0.84, 1.12	0.70
**Milk type used (reference: Full cream)**			
Never/rarely have milk	1.17	1.00, 1.37	0.06
Semi-skimmed	1.00	0.93, 1.08	0.99
Skimmed	1.02	0.94, 1.11	0.60
Soya	0.92	0.82, 1.04	0.20
Other type of milk	0.94	0.77, 1.15	0.53
**Spread type (reference: Butter/spreadable butter)**			
Flora Pro-Active/Benecol	0.39	0.28, 0.56	< 0.01
Never/rarely use spread	1.03	0.96, 1.10	0.39
Other type of spread/margarine	1.08	1.04, 1.12	0.00
**Bread type (reference: Brown)**			
White	0.98	0.92, 1.04	0.45
Wholemeal or wholegrain	0.85	0.81, 0.9	< 0.01
Other type of bread	0.87	0.79, 0.96	0.01
**Salt added to food (reference: Always)**			
Usually	0.92	0.83, 1.01	0.09
Sometimes	0.91	0.83, 1.00	0.05
Never/rarely	0.90	0.82, 0.98	0.02
**Coffee type (reference: Decaffeinated coffee)**			
Ground coffee	1.03	0.97, 1.09	0.29
Instant coffee	0.97	0.93, 1.02	0.24
Other type of coffee	1.22	1.08, 1.39	< 0.01
**Cereal type (reference: Biscuit cereal)**			
Bran cereal	0.93	0.88, 0.98	0.01
Muesli	0.88	0.83, 0.93	< 0.01
Oat cereal	0.92	0.87, 0.97	< 0.01
Other	1.09	1.03, 1.15	< 0.01
**Cereal intake**	1.00	0.99, 1.01	0.48
**Tea intake**	1.01	1.01, 1.02	< 0.01
**Coffee intake**	1.02	1.01, 1.03	< 0.01
**Water intake**	1.03	1.02, 1.04	< 0.01
**Cooked vegetable intake**	1.00	0.99, 1.01	0.41
**Salad raw vegetable intake**	0.99	0.98, 1	0.11
**Fresh fruit intake**	1.01	0.99, 1.02	0.27
**Dried fruit intake**	0.99	0.97, 1	0.01
**Bread intake**	1.00	1, 1	0.60

Model 1 was adjusted for age, sex, ethnic background, Townsend deprivation index, average household income, alcohol intake frequency and educational qualifications.

**Table 3.** Logistic regression results in model 2.

Variable	OR	95%CI	P
**Oily fish intake (reference: Never)**			
Less than once a week	0.87	0.81, 0.94	< 0.01
Once a week	0.87	0.81, 0.94	< 0.01
2–4 times a week	0.86	0.80, 0.93	< 0.01
5–6 times a week	0.79	0.62, 1.00	0.05
Once or more daily	1.01	0.67, 1.53	0.96
**Non oily fish intake (reference: Never)**			
Less than once a week	0.91	0.81, 1.01	0.09
Once a week	0.92	0.83, 1.03	0.15
2–4 times a week	0.98	0.87, 1.10	0.75
5–6 times a week	0.81	0.59, 1.11	0.19
Once or more daily	1.01	0.57, 1.79	0.98
**Processed meat intake (reference: Never)**			
Less than once a week	1.06	0.98, 1.16	0.15
Once a week	1.13	1.04, 1.24	0.01
2–4 times a week	1.16	1.06, 1.27	< 0.01
5–6 times a week	1.20	1.05, 1.37	0.01
Once or more daily	1.36	1.08, 1.71	0.01
**Poultry intake (reference: Never)**			
Less than once a week	1.03	0.91, 1.17	0.61
Once a week	0.99	0.88, 1.12	0.90
2–4 times a week	0.98	0.87, 1.10	0.76
5–6 times a week	1.06	0.89, 1.26	0.52
Once or more daily	0.98	0.66, 1.47	0.94
**Beef intake (reference: Never)**			
Less than once a week	1.01	0.94, 1.10	0.73
Once a week	0.97	0.89, 1.06	0.52
2–4 times a week	0.87	0.79, 0.96	< 0.01
5–6 times a week	0.79	0.48, 1.33	0.38
Once or more daily	1.52	0.75, 3.08	0.24
**Lamb mutton intake (reference**: Never)			
Less than once a week	1.11	1.05, 1.18	< 0.01
Once a week	1.19	1.11, 1.28	< 0.01
2–4 times a week	1.23	1.09, 1.39	< 0.01
5–6 times a week	0.92	0.35, 2.42	0.87
Once or more daily	0.47	0.13, 1.71	0.25
**Pork intake (reference: Never)**			
Less than once a week	0.87	0.82, 0.92	< 0.01
Once a week	0.93	0.87, 1.00	0.04
2–4 times a week	1.06	0.95, 1.19	0.27
5–6 times a week	0.29	0.09, 0.94	0.04
Once or more daily	3.60	1.63, 7.95	< 0.01
**Cheese intake (reference: Never)**			
Less than once a week	0.96	0.85, 1.08	0.50
Once a week	0.90	0.80, 1.01	0.08
2–4 times a week	0.88	0.79, 0.99	0.04
5–6 times a week	0.86	0.76, 0.98	0.02
Once or more daily	0.98	0.85, 1.13	0.77
**Milk type used (reference: Full cream)**			
Never/rarely have milk	1.14	0.98, 1.34	0.10
Semi-skimmed	0.98	0.91, 1.06	0.61
Skimmed	1.00	0.92, 1.08	0.96
Soya	0.91	0.81, 1.03	0.15
Other type of milk	0.92	0.75, 1.13	0.41
**Spread type (reference: Butter/spreadable butter)**			
Flora Pro-Active/Benecol	0.39	0.27, 0.55	< 0.01
Never/rarely use spread	1.03	0.97, 1.10	0.32
Other type of spread/margarine	1.08	1.04, 1.12	< 0.01
**Bread type (reference: Brown)**			
White	0.98	0.92, 1.04	0.41
Wholemeal or wholegrain	0.85	0.81, 0.90	< 0.01
Other type of bread	0.87	0.79, 0.96	0.01
**Salt added to food (reference: Always)**			
Usually	0.92	0.83, 1.01	0.10
Sometimes	0.91	0.83, 0.99	0.04
Never/rarely	0.90	0.82, 0.98	0.02
**Coffee type (reference: Decaffeinated coffee)**			
Ground coffee	1.04	0.98, 1.10	0.18
Instant coffee	0.98	0.93, 1.02	0.28
Other type of coffee	1.23	1.08, 1.40	< 0.01
**Cereal type (reference: Biscuit cereal)**			
Bran cereal	0.93	0.88, 0.99	0.01
Muesli	0.89	0.84, 0.94	< 0.01
Oat cereal	0.93	0.88, 0.98	0.01
Other	1.09	1.03, 1.16	< 0.01
**Cereal intake**	1.00	1.00, 1.01	0.37
**Tea intake**	1.02	1.01, 1.02	< 0.01
**Coffee intake**	1.02	1.01, 1.03	< 0.01
**Water intake**	1.03	1.02, 1.04	< 0.01
**Cooked vegetable intake**	1.00	0.99, 1.01	0.85
**Salad raw vegetable intake**	1.00	0.99, 1.01	0.55
**Fresh fruit intake**	1.01	1.00, 1.02	0.05
**Dried fruit intake**	0.99	0.98, 1.00	0.04
**Bread intake**	1.00	1.00, 1.00	0.77

Model 2 was adjusted for age, sex, ethnic background, Townsend deprivation index, average household income, educational qualifications, alcohol intake frequency, IPAQ groups and smoking status.

**Table 4.** Logistic regression results in model 3.

Variable	OR	95%CI	P
**Oily fish intake (reference: Never)**			
Less than once a week	0.87	0.81, 0.94	< 0.01
Once a week	0.88	0.81, 0.94	< 0.01
2–4 times a week	0.86	0.80, 0.94	< 0.01
5–6 times a week	0.78	0.61, 1.00	0.05
Once or more daily	1.03	0.68, 1.57	0.89
**Non oily fish intake (reference: Never)**			
Less than once a week	0.92	0.83, 1.03	0.17
Once a week	0.93	0.83, 1.04	0.21
2–4 times a week	0.98	0.88, 1.11	0.78
5–6 times a week	0.81	0.59, 1.12	0.20
Once or more daily	1.01	0.56, 1.81	0.98
**Processed meat intake (reference: Never)**			
Less than once a week	1.07	0.99, 1.17	0.10
Once a week	1.14	1.04, 1.24	< 0.01
2–4 times a week	1.17	1.07, 1.28	< 0.01
5–6 times a week	1.21	1.06, 1.39	0.01
Once or more daily	1.36	1.08, 1.71	0.01
**Poultry intake (reference: Never)**			
Less than once a week	1.04	0.92, 1.17	0.58
Once a week	0.99	0.88, 1.11	0.85
2–4 times a week	0.97	0.86, 1.09	0.60
5–6 times a week	1.03	0.87, 1.23	0.72
Once or more daily	0.92	0.61, 1.39	0.70
**Beef intake (reference: Never)**			
Less than once a week	1.01	0.93, 1.09	0.88
Once a week	0.95	0.87, 1.03	0.23
2–4 times a week	0.84	0.76, 0.92	< 0.01
5–6 times a week	0.79	0.47, 1.32	0.37
Once or more daily	1.36	0.66, 2.79	0.41
**Lamb mutton intake (reference: Never)**			
Less than once a week	1.12	1.06, 1.19	< 0.01
Once a week	1.20	1.12, 1.29	< 0.01
2–4 times a week	1.24	1.10, 1.40	< 0.01
5–6 times a week	0.91	0.34, 2.43	0.85
Once or more daily	0.43	0.11, 1.62	0.21
**Pork intake (reference: Never)**			
Less than once a week	0.88	0.83, 0.94	< 0.01
Once a week	0.95	0.88, 1.02	0.12
2–4 times a week	1.08	0.97, 1.21	0.17
5–6 times a week	0.30	0.09, 0.97	0.04
Once or more daily	3.95	1.77, 8.83	< 0.01
**Cheese intake (reference: Never)**			
Less than once a week	0.96	0.86, 1.09	0.54
Once a week	0.91	0.81, 1.03	0.14
2–4 times a week	0.92	0.82, 1.03	0.16
5–6 times a week	0.92	0.81, 1.04	0.20
Once or more daily	1.06	0.91, 1.22	0.47
**Milk type used (reference: Full cream)**			
Never/rarely have milk	1.11	0.95, 1.31	0.20
Other type of milk	0.91	0.74, 1.11	0.34
Semi-skimmed	0.98	0.91, 1.05	0.55
Skimmed	0.98	0.90, 1.06	0.59
Soya	0.92	0.81, 1.04	0.16
**Spread type (reference: Butter/spreadable butter)**			
Flora Pro-Active/Benecol	0.37	0.26, 0.53	< 0.01
Never/rarely use spread	1.00	0.94, 1.07	0.89
Other type of spread/margarine	1.05	1.01, 1.10	0.01
**Bread type (reference: Brown)**			
Other type of bread	0.88	0.80, 1.01	0.02
White	0.97	0.91, 0.98	0.34
Wholemeal or wholegrain	0.87	0.82, 1.03	< 0.01
**Salt added to food (reference: Always)**			
Never/rarely	0.90	0.82, 0.98	0.02
Sometimes	0.90	0.82, 0.99	0.03
Usually	0.92	0.83, 1.01	0.09
**Coffee type (reference: Decaffeinated coffee)**			
Ground coffee	1.05	1.00, 1.11	0.07
Instant coffee	0.96	0.92, 1.01	0.09
Other type of coffee	1.22	1.07, 1.39	< 0.01
**Cereal type (reference: Biscuit cereal)**			
Bran cereal	0.94	0.88, 0.99	0.03
Muesli	0.91	0.86, 0.97	< 0.01
Oat cereal	0.94	0.89, 0.99	0.02
Other	1.08	1.02, 1.14	0.01
**Cereal intake**	1.01	1.00, 1.02	0.01
**Tea intake**	1.02	1.01, 1.02	< 0.01
**Coffee intake**	1.02	1.01, 1.03	< 0.01
**Water intake**	1.04	1.03, 1.05	< 0.01
**Cooked vegetable intake**	1.00	0.99, 1.01	0.71
**Salad raw vegetable intake**	1.00	0.99, 1.01	0.45
**Fresh fruit intake**	1.01	1.00, 1.03	0.01
**Dried fruit intake**	0.99	0.98, 1.00	0.10
**Bread intake**	1.00	1.00, 1.01	0.02

Model 3 was adjusted for age, sex, ethnic background, Townsend deprivation index, average household income, educational qualifications, alcohol intake frequency, IPAQ groups, smoking status, body mass index (BMI), weight, and standing height.

The results of sensitivity analysis are displayed in Table S1-S2. Generally, the standardized VIF for the diet-related variables was significantly less than 10, indicating that there was no apparent collinearity among these variables. Otherwise, the significance of each dietary factor in the subsample analysis was basically consistent with that observed in the main analysis. However, some results from the sub-sample analysis differ from those obtained in the primary logistic model. For instance, when compared to Model 3, variables such as consuming pork 5–6 times a week, all frequencies of salt intake, a preference for “other” categories in cereal intake, and weekly bread intake did not exhibit significant associations with sarcopenia. These subtle discrepancies might arise from sample-specific biases; however, they do not impact the main conclusions drawn in the subsequent discussion.

After incorporating ALM into the diagnostic criteria, several food intake frequencies were significantly associated with confirmed sarcopenia (Table 5, reference: never): Non-oily fish intake showed an inverse association, with OR values of (0.60, 0.41–0.88, *p* < 0.01) for less than once a week, (0.62, 0.42–0.91, *p* = 0.01) for once a week, and (0.59, 0.4–0.89, *p* = 0.01) for 2–4 times a week. Conversely, processed meat intake was positively associated with sarcopenia, with OR values of (1.42, 1.1–1.86, *p* < 0.01) for less than once a week, (1.40, 1.06–1.86, *p* = 0.02) for once a week, (1.75, CI: 1.3–2.35, *p* < 0.01) for 2–4 times a week, and (1.97, 1.14–3.28, *p* = 0.01) for 5–6 times a week. Lamb mutton intake 2–4 times a week also showed a positive association (1.75, 1.11–2.7, *p* = 0.01). Additionally, certain food subgroups were correlated with sarcopenia risk. Wholemeal or wholegrain bread (0.71, 0.59–0.86, *p* < 0.01), rarely or never adding salt to food (0.64, 0.47–0.9, *p* < 0.01), and bran cereal (0.73, 0.58–0.92, *p* < 0.01) were all inversely associated with sarcopenia. Lastly, specific weekly intakes of water (1.06, 1.03–1.09, *p* < 0.01), dried fruit (0.94, 0.9–0.98, *p* < 0.01), and bread (0.99, 0.98–1, *p* = 0.01) were also associated with sarcopenia (Table 5).

**Table 5.** Logistic regression results of confirmed sarcopenia.

Variable	OR	95%CI	P
**Oily fish intake (reference: Never)**			
Less than once a week	1.16	0.86, 1.59	0.33
Once a week	1.17	0.87, 1.6	0.31
2–4 times a week	1.05	0.76, 1.46	0.77
5–6 times a week	0.88	0.3, 2.09	0.78
Once or more daily	0.97	0.05, 5.23	0.98
**Non oily fish intake (reference: Never)**			
Less than once a week	0.60	0.41, 0.88	< 0.01
Once a week	0.62	0.42, 0.91	0.01
2–4 times a week	0.59	0.4, 0.89	0.01
5–6 times a week	0.63	0.18, 1.67	0.40
Once or more daily	0.13	0, 1.68	0.21
**Processed meat intake (reference: Never)**			
Less than once a week	1.42	1.1, 1.86	< 0.01
Once a week	1.40	1.06, 1.86	0.02
2–4 times a week	1.75	1.3, 2.35	< 0.01
5–6 times a week	1.97	1.14, 3.28	0.01
Once or more daily	2.28	0.85, 5.21	0.07
**Poultry intake (reference: Never)**			
Less than once a week	0.82	0.56, 1.2	0.30
Once a week	0.84	0.58, 1.22	0.34
2–4 times a week	0.85	0.59, 1.24	0.39
5–6 times a week	1.52	0.84, 2.67	0.15
Once or more daily	0.97	0.14, 3.99	0.96
**Beef intake (reference: Never)**			
Less than once a week	0.88	0.68, 1.13	0.30
Once a week	0.90	0.69, 1.19	0.45
2–4 times a week	0.81	0.59, 1.12	0.21
5–6 times a week	0.93	0.05, 4.8	0.94
Once or more daily	0.69	0, 2.72	0.98
**Lamb mutton intake (reference: Never)**			
Less than once a week	1.14	0.92, 1.43	0.22
Once a week	1.15	0.9, 1.48	0.27
2–4 times a week	1.75	1.11, 2.7	0.01
5–6 times a week	0.79	0.41, 1.38	0.98
Once or more daily	0.81	0.50, 1.72	0.98
**Pork intake (reference: Never)**			
Less than once a week	0.97	0.79, 1.21	0.80
Once a week	0.98	0.77, 1.26	0.87
2–4 times a week	0.94	0.58, 1.46	0.77
5–6 times a week	0.57	0.18, 1.23	0.98
Once or more daily	4.33	0.26, 9.48	0.15
**Cheese intake (reference: Never)**			
Less than once a week	1.06	0.69, 1.7	0.81
Once a week	1.08	0.71, 1.73	0.72
2–4 times a week	1.09	0.72, 1.72	0.71
5–6 times a week	0.85	0.54, 1.39	0.49
Once or more daily	0.84	0.5, 1.44	0.51
**Milk type used (reference: Full cream)**			
Never/rarely have milk	1.44	0.86, 2.34	0.15
Other type of milk	0.59	0.24, 1.25	0.21
Semi-skimmed	1.00	0.78, 1.29	0.99
Skimmed	1.11	0.84, 1.48	0.46
Soya	1.22	0.85, 1.76	0.27
**Spread type (reference: Butter/spreadable butter)**			
Flora Pro-Active/Benecol	0.30	0.05, 0.97	0.09
Never/rarely use spread	1.04	0.83, 1.31	0.70
Other type of spread/margarine	1.10	0.96, 1.27	0.17
**Bread type (reference: Brown)**			
Other type of bread	0.91	0.66, 1.25	0.58
White	0.90	0.72, 1.13	0.35
Wholemeal or wholegrain	0.71	0.59, 0.86	< 0.01
**Salt added to food (reference: Always)**			
Never/rarely	0.64	0.47, 0.9	< 0.01
Sometimes	0.73	0.53, 1.02	0.05
Usually	0.91	0.65, 1.3	0.60
**Coffee type (reference: Decaffeinated coffee)**			
Ground coffee	0.91	0.75, 1.11	0.34
Instant coffee	0.97	0.83, 1.14	0.73
Other type of coffee	1.49	0.96, 2.23	0.06
**Cereal type (reference: Biscuit cereal)**			
Bran cereal	0.73	0.58, 0.92	< 0.01
Muesli	0.89	0.73, 1.09	0.26
Oat cereal	0.87	0.72, 1.06	0.17
Other	0.98	0.79, 1.23	0.89
**Cereal intake**	0.99	0.96, 1.02	0.58
**Tea intake**	1.02	0.99, 1.04	0.25
**Coffee intake**	0.98	0.94, 1.02	0.29
**Water intake**	1.06	1.03, 1.09	< 0.01
**Cooked vegetable intake**	1.01	0.97, 1.04	0.71
**Salad raw vegetable intake**	0.98	0.95, 1.02	0.33
**Fresh fruit intake**	1.02	0.98, 1.06	0.30
**Dried fruit intake**	0.94	0.9, 0.98	< 0.01
**Bread intake**	0.99	0.98, 1	0.01

### 3.3. Associations between dietary factors and sarcopenia related traits in the MR analysis

**Figure 1.**
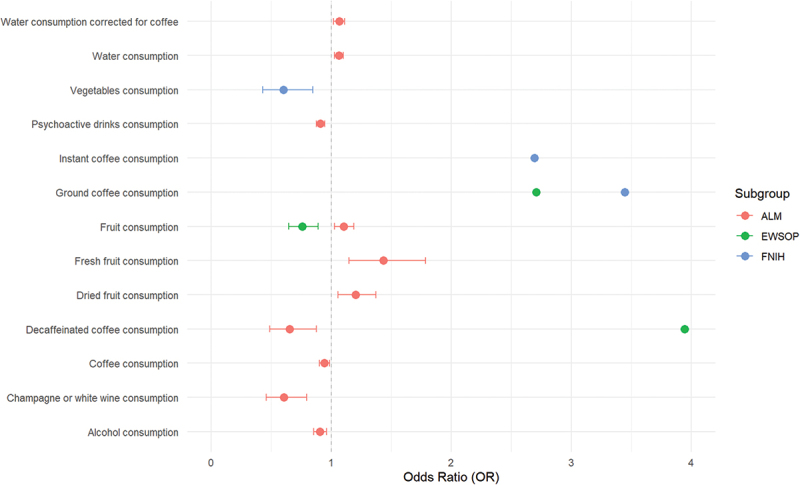
MR results of dietary factors and sarcopenia related traits.

To further detect causal relationship, MR was conducted between dietary factors and sarcopenia traits, including low hand grip strength, based on two criteria, and ALM (Figure 1). Firstly, we employed IVW method to identify the causal effects of various eating habits on sarcopenia-related phenotypes. Vegetable consumption and coffee consumption had a significant causal relationship with low hand grip strength according to FNIH definition, while fruit consumption and coffee consumption had a significant causal relationship with low hand grip strength according to EWGSOP definition. MR showed that low hand grip strength defined by FNIH was mitigated by vegetables consumption (OR, 0.60; 95% CI, 0.45 to 0.84, *p* < 0.0035), and aggravated by decaffeinated coffee consumption (OR, 5.03; 95% CI, 1.71 to 14.83, *p* < 0.0033), ground coffee consumption (OR, 3.45; 95% CI, 1.50 to 7.90, *p* < 0.0034), and instant coffee consumption (OR, 2.69; 95% CI, 1.34 to 5.41, *p* < 0.005). Besides, low hand grip strength by EWGSOP was mitigated by fruit consumption (OR, 0.76; 95% CI, 0.64 to 0.89, *p* < 0.001), and aggravated by decaffeinated coffee consumption (OR, 3.95; 95% CI, 1.85 to 8.42, *p* < 0.0003) and ground coffee consumption (OR, 2.71; 95% CI, 1.52 to 4.83, *p* < 0.0007). As for ALM, MR showed that alcohol (OR, 0.91; 95% CI, 0.85 to 0.96, *p* < 0.001), coffee (OR, 0.94; 95% CI, 0.90 to 0.99, *p* < 0.008) and psychoactive drinks consumption (OR, 0.91; 95% CI, 0.88 to 0.94, *p* < 0.0001) decreased ALM. Furthermore, decaffeinated coffee (OR, 0.65; 95% CI, 0.49 to 0.88, *p* < 0.004) and champagne or white wine consumption (OR, 0.60; 95% CI, 0.46 to 0.80, *p* < 0.0003) showed particularly significant negative effects on ALM. On the contrary, fruit (OR, 1.11; 95% CI, 1.03 to 1.19, *p* < 0.006), dried fruit (OR, 1.20; 95% CI, 1.06 to 1.37, *p* < 0.004), fresh fruit (OR, 1.43; 95% CI, 1.15 to 1.79, *p* < 0.001) and water consumption (OR, 1.06; 95% CI, 1.03 to 1.10, *p* < 0.0003) increased ALM.

Effects of different food intake on Low hand grip strength (HGS), appendicular lean mass (ALM). Red, green and blue represent the effects of the same food on ALM, low HGS diagnosed by European Working Group on Sarcopenia in Older People (EWSOP) and Foundation for the National Institutes of Health (FNIH), respectively.

**Figure 2.**
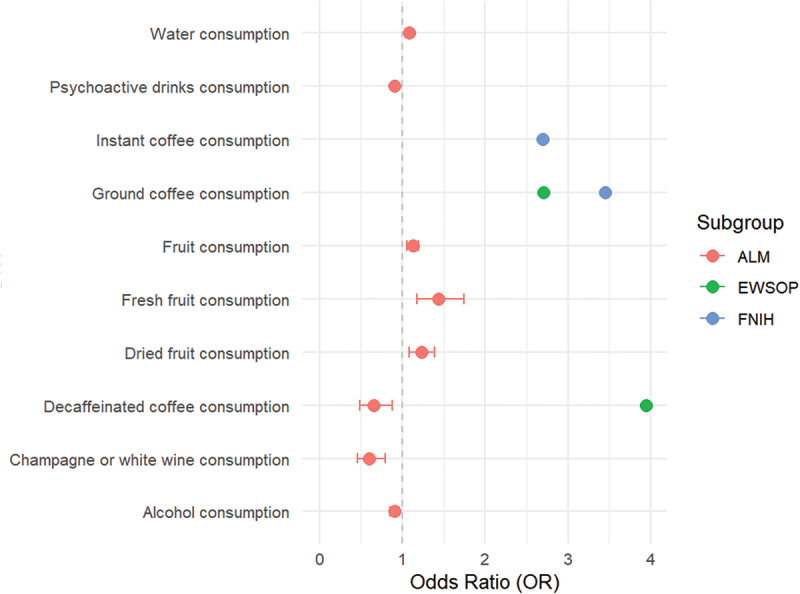
MR analysis results after screening heterogeneity and pleiotropy.

Subsequently, sensitivity analysis found partial heterogeneity and horizontal pleiotropy among the above results (Table S3-S4). The causal effects of fruit intake on low grip strength diagnosed by EWSOP and water consumption, fresh fruit consumption, water consumption corrected for coffee, dried fruit consumption, fruit consumption and coffee consumption on ALM showed significant heterogeneity while the causal effect of vegetable intake on FNIH showed obvious horizontal pleiotropy. Therefore, we reevaluated the relationship between these dietary factors and sarcopenia based on the principles described in methods by weighted median and MR-egger. The results indicated that only water consumption, fresh fruit consumption, dried fruit consumption and fruit consumption still had significant effects on ALM after method adjustment (Figure 2). The statistical power of the instrumental variants finally included in the MR analysis was displayed in supplementary materials.

Effects of different food intake on Low hand grip strength (HGS), appendicular lean mass (ALM). Red, green and blue represent the effects of the same food on ALM, low HGS diagnosed by European Working Group on Sarcopenia in Older People (EWSOP) and Foundation for the National Institutes of Health (FNIH), respectively. According to the description in methods, the weighted median method was adopted for the variable with heterogeneity.

## 4. Discussion

In this study, we initially conducted a cross-sectional analysis using a mutivariable logistic regression model in the UK Biobank population, identifying associations between various dietary factors and probable sarcopenia, as diagnosed by grip strength. Sensitivity analyses confirmed the robustness of these findings. Furthermore, in the analysis of confirmed sarcopenia using the combined diagnostic criteria of hand grip strength and ALM, the effects of certain dietary factors were observed to change. The potential mechanisms underlying these changes will be discussed in a later section. Subsequently, to substantiate these findings at a causal level, we utilized MR analysis to pinpoint the genetic association between these dietary factors and sarcopenia-related phenotype, including low grip strength (defined by EWGSOP and FNIH standards) and ALM.

The weekly intake frequency of various food was identified as being associated with probable sarcopenia in logistic regression models. Specifically, intake of oily fish was associated with a reduced risk of sarcopenia across three models, with the effect as estimated by OR, becoming more pronounced with increased frequency of consumption. In contrast, intake of non-oily fish did not show a significant association with sarcopenia. This discrepancy may be attributed to the higher content of fish oil in oily fish compared to non-oily fish, providing greater amounts of unsaturated fatty acids such as omega-3. Previous studies have demonstrated that omega-3 fatty acids can increase muscle mass and enhance muscle function, thereby reducing the risk of sarcopenia and slowing the progression of this condition [34,35]. Interestingly, when the intake frequency of oily fish increased to 5–6 times per week or more, the significance of its association with sarcopenia began to diminish. At the highest frequency of intake, once or more daily, the association became insignificant across all three models and sensitivity analyses. The underlying cause of this phenomenon remains unclear and warrants further investigation. Otherwise, the consumption of processed meat was consistently identified as a risk factor for sarcopenia in all logistic regression models. An increase in consumption frequency, starting from as little as once per week, was linked to a proportional rise in the magnitude of its effect. Previous epidemiological studies have suggested that higher intake of processed meats [36] was associated with an increased risk of various chronic diseases [37,38]. In this study, we firstly established a direct and robust connection between processed meat consumption and risk of sarcopenia. Additionally, the frequency of lamb consumption, when it ranges from less than once per week to 2–4 times per week, may increase the risk of sarcopenia. Conversely, reducing the frequency of salt intake may be associated with decreased risk of sarcopenia in main models, but this effect became insignificant in sensitivity analysis. The impact of the aforementioned types of foods on the risk of sarcopenia demonstrated a consistent effect trend with variations in consumption frequency. However, for many other foods, including beef, pork, although certain effects were observed at specific frequencies compared to never being consumed, these effects were not stable across different models and sensitivity analysis and did not exhibit a consistent trend with incremental changes in intake frequency. In some cases, certain foods even displayed opposite effects as their consumption frequency increases, which made it challenging to draw clinically valuable conclusions based on these analytical results.

In view of the increasingly refined food types, in addition to discovering the association between intake frequency and sarcopenia, it is also important to determine the impact of different types of the same food group on sarcopenia. Our study indicated that in spread type, Flora Pro-Active/Benecol were significantly associated with reduced risk of sarcopenia compared to butter. Otherwise, wholemeal bread, Bran/Oat/Muesli cereal also reduced the risk of sarcopenia compared to brown bread/Biscuit cereal.

In our analysis, specific dietary factors characterized by certain weekly intake amounts, in addition to intake frequency and food subtypes, demonstrated associations with sarcopenia. For example, the consumption of water, coffee, and tea was associated with a slightly increased risk of sarcopenia. Previous studies have generally suggested an association between higher coffee consumption and an increased risk of reduced muscle strength and mass [39,40], which aligned with our findings. However, some studies indicated that green tea consumption might offer protective effects against sarcopenia [41], while our study included both green and black tea in its tea consumption variable. This difference may account for the inconsistent results observed. Notably, the association between water intake and sarcopenia was identified for the first time in this study, and the underlying mechanisms require further investigation in future research. Furthermore, fresh fruit intake did not influence the risk of sarcopenia diagnosed by HGS in Model 1, but it marginally increased the risk in Models 2 and 3. This finding contrasted with previous studies, which suggested that fruit intake could reduce the risk of sarcopenia. Consequently, due to the heterogeneity observed in the outcomes of different models within the logistic analysis and the opposite results of the previous study, it was challenging to draw a consistent conclusion regarding the risk of sarcopenia associated with fresh fruit intake based on the cross-sectional analysis. Additionally, dried fruit consumption was associated with a modest reduction in the risk of sarcopenia in Models 1 and 2. After adjusting for body mass index (BMI) and standardizing for weight and height, the P-value was no longer significant, indicating that the impact of dried fruit intake on sarcopenia may be modulated by factors such as BMI.

Besides the associations between diet and sarcopenia revealed by cross-sectional analysis, MR analysis identified causal relationships between multiple dietary factors and low HGS and appendicular lean mass (ALM). Decaffeinated coffee consumption was observed to significantly increase the risk of low HGS in two diagnostic criteria and reduce the ALM. Conversely, ground coffee consumption was found to significantly increase the risk based on the FNIH standard for low grip strength. Notably, ground coffee and instant coffee consumption were associated with a slight reduction in the risk of low grip strength when evaluated against the EWSOP and FNIH standards, respectively. However, given that the OR values for these effects were very close to 1, the observed protective effects should be interpreted with caution. Furthermore, the analysis of ground coffee consumption yielded contradictory results under the two grip strength criteria, suggesting that the protective effects of these two types of coffee on low grip strength may not be consistent. Otherwise, the intake of fresh fruit, dried fruit, and fruit in general (without distinguishing specific types) was shown to significantly increase ALM, thus we can infer that fruit intake may have a protective effect against sarcopenia. Otherwise, while alcohol intake did not demonstrate a causal association with the two criteria for low HGS in the MR analysis, consumption of champagne or white wine was found to significantly reduce ALM. The risk effect of these specific beverages on ALM notably exceeded the impact of overall alcohol consumption on ALM (OR: 0.60 vs 0.90). Such a divergence may be accounted for by distinct compounds contained in champagne and white wine, including specific organic acids and polyphenols – that may directly affect muscle metabolism more adversely than ethanol alone. Therefore, in addition to reducing overall alcohol consumption, minimizing the intake of champagne or white wine should potentially be considered for individuals with sarcopenia. Furthermore, psychoactive drinks consumption was found to reduce ALM in the analysis, while Water consumption was found to increase ALM. The detrimental effects of psychoactive drinks on ALM was likely attributable to the psychoactive substances they contain, such as caffeine and ethanol, which was also consistent with the previously mentioned analysis results of alcohol and caffeine.

Although our study identified several dietary factors significantly associated with the risk of sarcopenia or its related phenotypes – through cross-sectional analyses under different diagnostic criteria (for probable and confirmed sarcopenia) and through MR analyses of handgrip strength and ALM – these associations exhibited considerable variation across different analyses and diagnostic criteria. The potential reasons for these discrepancies can be explained from multiple perspectives. For example, oily fish, which showed a significant inverse association with the risk of probable sarcopenia across multiple models, did not demonstrate a significant association with confirmed sarcopenia in the analysis. The differential associations may stem from distinct biological mechanisms and methodological factors. ω-3 fatty acids likely improve neuromuscular function [42] and reduce inflammation [43], more effectively attenuating grip strength decline – explaining the stronger association with probable sarcopenia. In contrast, muscle mass (ALMI) maintenance involves long-term protein balance and is influenced by multiple factors such as hormones, exercise, and overall nutrition [44,45]. ω-3 effects on muscle mass may require longer exposure or synergism with other nutrients, which cross-sectional designs cannot adequately capture. Furthermore, probable sarcopenia represents an early, nutrition-responsive stage, while confirmed sarcopenia indicates advanced progression with potentially irreversible loss. ALMI is also more susceptible to confounding by weight, physical activity, and metabolic rate, whereas grip strength is less affected by these factors and may better reflect direct nutritional influences. Additionally, water intake was found to have a slight positive association with the risk of both probable and confirmed sarcopenia. However, in the MR analysis, it was associated with increased ALM. The differing results are likely due to methodological differences and possible reverse causality. Mendelian randomization (MR) uses genetic variants to infer causality, which helps minimize reverse causation and confounding. The positive MR finding indicates that higher lifelong water intake may genuinely support muscle accumulation. In contrast, cross-sectional studies can be influenced by reverse causation – for example, people already diagnosed with sarcopenia may intentionally drink more water. This highlights that statistical associations should not be directly interpreted as clinically meaningful. The variation in findings underscores the need for careful interpretation based on biological context. Taking both MR and observational evidence into account, we propose that long-term increases in water intake might have a small beneficial effect on muscle mass. This phenomenon may be due to water intake’s influence on energy expenditure and fat oxidation, thereby contributing to improved body composition. However, whether this effect is practically meaningful in clinical practice remains uncertain and may require further validation through dedicated cohort studies or mechanistic research.

Nevertheless, there were some limitations to our study. Firstly, our study employed a cross-sectional design for the observational analysis, rather than a longitudinal cohort study, thus limiting our ability to thoroughly explore the causal relationship between diet and sarcopenia using real-world data. Participants’ baseline dietary factors are challenging to maintain consistently over many years, prompting our reliance on cross-sectional data to avoid biases from dietary changes over a long-time span. Therefore, cohort studies capable of obtaining real-time dietary data through long-term, periodic follow-ups are crucial for eliminating this bias and further exploring the impact of diet on sarcopenia. Secondly, the cross-sectional analyses showed heterogeneous results between probable sarcopenia and confirmed sarcopenia. Although these differences can be partially explained from multiple perspectives, further cohort studies or biological investigations are needed to fully elucidate the underlying causes.

In conclusion, this study employed both multivariable logistic regression and MR analyses to explore the associations between dietary factors and sarcopenia in the UK Biobank population. Cross-sectional analyses revealed several significant associations, such as the protective effect of oily fish and whole-grain products against probable sarcopenia, and the adverse effect of processed meat. Notably, some associations varied between probable and confirmed sarcopenia, highlighting the influence of diagnostic criteria and underlying mechanisms. MR analysis further provided genetic evidence supporting causal roles of specific dietary factors – such as decaffeinated coffee, fruit intake, and alcohol subtypes – in influencing grip strength and ALM. Importantly, discrepancies between observational and MR findings underscore the complexities of diet-sarcopenia relationships and the limitations of cross-sectional designs, including potential reverse causality. Despite these insights, the heterogeneous results emphasize the need for cautious interpretation and further validation through longitudinal cohorts and mechanistic studies. This research contributes to a deeper understanding of the modifiable dietary risk factors for sarcopenia and informs potential nutritional strategies for prevention, while also highlighting the necessity of more robust study designs to clarify causal pathways and clinical relevance.

## Data Availability

The complete individual level data used in this study were obtained by researchers from the UK Biobank through paid application (under the application number 95,082), and can only be used in a specific research scope, so they cannot be completely shared. To access these individual levels, please refer to the website of UK Biobank (http://www.ukbiobank.ac.uk/) through official application. For the result data generated in the analysis of this study, please contact the author by this e-mail address (jeeven_chen@163.com).
